# ^13^C-DNA-SIP Distinguishes the Prokaryotic Community That Metabolizes Soybean Residues Produced Under Different CO_2_ Concentrations

**DOI:** 10.3389/fmicb.2019.02184

**Published:** 2019-09-24

**Authors:** Yanhong Wang, Zhenhua Yu, Yansheng Li, Guanghua Wang, Caixian Tang, Xiaobing Liu, Junjie Liu, Zhihuang Xie, Jian Jin

**Affiliations:** ^1^Key Laboratory of Mollisols Agroecology, Northeast Institute of Geography and Agroecology, Chinese Academy of Sciences, Harbin, China; ^2^Centre for Experiment, Guizhou University of Traditional Chinese Medicine, Guiyang, China; ^3^Centre for AgriBioscience, La Trobe University, Bundoora, VIC, Australia

**Keywords:** black soil, carbon cycle, crop residue, isopycnic centrifugation, Miseq sequencing

## Abstract

The amendment of crop residues produced under elevated CO_2_ (eCO_2_) may alter soil microbial community structure and their functions on residue decomposition and carbon (C) cycling in soil. The key to understanding this process is to elucidate the structure of prokaryotic communities that metabolize crop residues derived from eCO_2_. A soil incubation experiment was conducted to explore the response of soil microbial community to the amendment of ^13^C-labeled soybean residues produced under ambient CO_2_ (aCO_2_) and eCO_2__._ The residues were applied to a Mollisol, followed by ^13^C-DNA stable isotope probing (SIP) and Illumina sequencing on soil prokaryotic community over time. The structure of residue-metabolizing community differed in response to the amendment of eCO_2_- and aCO_2_-derived residues after 28 days of incubation. In particular, genera *Actinomadura*, *Nocardia*, *Non-omuraea*, and *Shimazuella* were the dominant members of the residue-metabolizing bacteria, which contributed to this difference. The relative abundances of genera *Actinomadura*, *Nocardia* and *Shimazuella* were 118–144%, 71–113%, and 2–4-fold higher in the Mollisol amended with aCO_2_-derived than eCO_2_-derived residue. In contrast, the relative abundance of *Non-omuraea* was 87–90% greater in the eCO_2_-residue treatment. However, during the incubation period, there was no difference between the two residue treatments in the community structure as a whole without SIP. These results implied that a pioneering prokaryotic community metabolized the residue initially prior to the entire community. Those bacteria genera being inhibited with the amendment of the eCO_2_-derived residue, compared to aCO_2_-derived residue, were likely preferential to metabolize recalcitrant C, which might be associated with changes of chemical composition of the residue under eCO_2_.

## Introduction

Atmospheric CO_2_ concentration has rapidly risen after the Industrial Revolution and currently exceeds 400 ppm^1^. It is predicted to continuously increase in the coming decades (Nowak et al., 2004). Elevated CO_2_ (eCO_2_) may affect plant biomass production (Ainsworth and Long, 2005; Ma et al., 2009), and residue chemical quality (Gifford et al., 2000). A number of studies reported that eCO_2_ increased lignin concentration and the carbon (C)-to-nitrogen (N) ratio (C/N ratio) in plants (Torbert et al., 2000; Norby et al., 2001; Sayer et al., 2011). A free-air CO_2_-enrichment (FACE) study showed that eCO_2_ (600 ppm) increased the C/N ratio in wheat and rice grown in Typic Haplustept (Viswanath et al., 2010). Cotrufo and Ineson (2000) also found that eCO_2_ increased C/N and lignin/N ratios by 59 and 37%, respectively, in beech twigs (*Fagus sylvatica*).

The eCO_2_-induced change in residue chemistry may considerably impact sequestration of soil organic C (SOC) in natural and agricultural ecosystems (van Groenigen et al., 2014; Wang et al., 2017). Williams et al. (2000) found that the eCO_2_ increased C accumulation in the physically protected SOC in a native prairie in Kansas. Moreover, Ma et al. (2009) reported inputs of wheat straw grown under eCO_2_ increased the SOC in a fluvo-aquic soil. However, understanding the contribution of eCO_2_-derived residue to SOC needs to reveal the microbial community structure and function that control both the SOC decomposition and stability (Sulman et al., 2014).

The decomposability of eCO_2_-derived plant residue would greatly depend on the responses of soil microbes. This is because the decomposition of plant residue in soil is primarily driven by microorganisms (Radajewski et al., 2000), and residue properties strongly influence the metabolisms and structure of the microbial community (Rui et al., 2009; Semenov et al., 2012; Pascault et al., 2013). In a 200 days incubation study, the amendment of the eCO_2_-derived wheat shoot into a Mollisol enriched the genera *Bryobacter*, *Candidatus Solibacter*, and *Gemmatimonas* compared to the amendment of the residue derived from ambient CO_2_ (aCO_2_), while suppressed *Actinomadura*, *Streptomyces*, and *Arthrobacter* (Wang et al., 2017). This change in the microbial community structure was associated with the residue contribution to soil C sequestration. However, to our knowledge, the information on the specific prokaryotic community involved in the decomposition of eCO_2_-derived soybean residue is limited. Considering that soybean is a major crop grown across the world and has lower C/N ratios in its residue than non-legume crops (Lian et al., 2017), an investigation is crucial to gain the insight of the residue-C sequestration in soil under climate change.

Previous molecular approaches such as phospholipid fatty acid (PLFA) analysis (Drenovsky et al., 2004; Baumann et al., 2009), DNA cloning and denaturing gradient gel electrophoresis (DGGE) (Lee et al., 2012; Fan et al., 2014) were commonly deployed to describe the dynamics of genetic structure and taxonomic composition of prokaryotic populations in the residue-amended soils in agricultural systems. However, these traditional approaches cannot directly probe the soil microbes which assimilate plant-residue C. Stable isotope probing (SIP) technology can be used to address this issue (Radajewski et al., 2000). Pascault et al. (2013) recently found that Firmicutes dominantly metabolized wheat residue in soil, while Proteobacteria were the major phylum metabolizing alfalfa residue.

This study used the SIP technique to compare the changes in the structure and diversity of prokaryotic communities that were involved in the decomposition of soybean residues produced under aCO_2_ and eCO_2_ environments. We hypothesized that the prokaryotic community assimilating the soybean residues would considerably differ because the chemistry of residual sources was changed when the plants were cultivated under different CO_2_ concentrations.

## Materials and Methods

### Residue and Soil Preparation

The soil used in this experiment was collected from top 10 cm tillage layer of a cropping paddock in Hailun (126.4°E, 47.3°N), Heilongjiang Province in northeast China. The soil type is Mollisols or Phaeozem (FAO-UNESCO, 1974). The soil was air-dried and sieved through a 2 mm mesh. The visible straw in the soil was manually removed. The air-dried soil was pre-incubated at 40% of field water capacity for 14 days at 25°C (Butterly et al., 2016).

The ^13^C-labeled soybean shoot residues used in this study were produced under aCO_2_ (390 ppm, aCO_2_-derived shoot) or eCO_2_ (550 ppm, eCO_2_-derived shoot). This eCO_2_ concentration is predicted to be reached by the middle of this century (de Graaff et al., 2006; Ainsworth et al., 2008). The soybean cultivar was Suinong 14, which is widely cultivated in northeast China. Seven uniform-sized seeds were sown in each pot containing 3 kg soil, and seedlings were thinned to two per pot 7 days after germination. Plants were grown in open-top chambers (OTC), supplying CO_2_ to designated levels. The OTC construction and the CO_2_ regulation were described in Li et al. (2017). The ^13^CO_2_ labeling was applied from the third node stage (V_3_) to the initial flowering stage (R_1_). Plants were labeled for 8 h daily in air-tight clear chambers with respective CO_2_ concentrations. The labeling time covered most of the plant photosynthetic period during the day. H_2_SO_4_ (9 M) was injected into Na_2_^13^CO_3_ (≥99.8 atom%, Sigma-Aldrich, St Louis, MO, United States) every 90 min to maintain the CO_2_ concentration at either 390 ppm or 550 ppm in chambers. The frequency of this injection was determined by setting up non-labeling controls under the same condition except for using Na_2_^12^CO_3_ instead of Na_2_^13^CO_3_ and measuring the decrease rate of the ^12^CO_2_ concentration for each CO_2_ treatment (Yu et al., 2017). Once the labeling event finished each day, plants were put back to OTCs for the rest of the day. After labeling for 25 days, plants were then harvested. Shoots including leaves and stems were oven-dried at 70°C for 72 h, and ground to 0.1–1 mm. The C and N concentrations of shoots were determined using an EL III Elemental Analyzer (Hanau, Germany). An acid-detergent method was used to determine the concentrations of lignin and cellulose in shoots (Goering and van Soest, 1970). The ^13^C enrichment of the shoot residue was measured using an isotope ratio mass spectrometer (Deltaplus, Finnigan MAT GmbH, Bremen, Germany).

### Experimental Setup

This experiment consisted of two residue treatments and three sampling times in a randomized complete block design. The two residues were the shoots of soybean that were produced under aCO_2_ and eCO_2_, respectively, as described above. There were three replicates for each sampling time. Thus, each residue treatment had total nine microcosms. On Days 7, 14, and 28 of the incubation, three microcosms from each treatment were randomly sampled as three replicates. Each microcosm comprised three compartments with residues in the central compartment and soil in the two side compartments (Supplementary Figure S1). This three-compartment design was to ensure that residues was surrounded by the soil on each side. Each compartment was made of a 1 mm-thick PVC frame with a hole of 3.2 cm in diameter in the middle, and this hole was covered with a 53 μm nylon mesh, which allowed microorganisms to move freely across the compartments. The shoot residue (0.3 g) was loaded into the central compartment and 1.0 g of soil was put into each side compartment. Then, the three frames were tightly clamped together to ensure residues firmly contacting soil. The clamped PVC frames were buried with 70 g of soil. With the design of the 1 mm-thick of each compartment, the influence of soil microbes on the decomposition of the amended residue was likely more uniform compared to the previous litter-bag technique (Viswanath et al., 2010). Soil moisture was maintained at 80% of field capacity (28% w/w) by watering the soil to the target weight every second day throughout the incubation period. The microcosms were incubated at 25°C in dark. At each sampling time, the soils and residues in microcosm compartments were collected in autoclaved Eppendorf tubes and immediately frozen in liquid nitrogen and then stored in −80°C before the DNA extraction.

### Soil DNA Extraction and Isopycnic Centrifugation

Soil DNA in each compartment of the microcosm was extracted from frozen samples (0.5 g) using the FastDNA Spin Kit for Soil (MP Biomedicals; Solon, OH, United States) combined with FastPrep-24 instrument (MP Biomedicals). The quality of DNA was determined by a NanoDrop 2000 Spectrophotometer (Bio-Rad Laboratories Inc., Hercules, CA, United States) and the integrity was checked by 1% (wt/vol) gel electrophorosis.

As the responses of microbial activity to crop residue amendment usually stabilize within 30 days (Nguyen et al., 2016), the samples collected at Days 7 and 28 were chosen for isopycnic centrifugation. DNA in the central compartment of the microcosm was extracted for three times, and the three DNA extracts were pooled to yield sufficient DNA for the separation of ^13^C-DNA. DNA extracts from the pre-incubated soil were referred to the non-labeled control and were ultra-centrifuged with the ^13^C-DNA in pair (Sul et al., 2009). Briefly, 5,000 ng DNA of each sample was loaded with cesium trifluoroacetate (CsTFA, Sigma, United States) and gradient buffer (0.1 M Tris HCl, 0.1 M KCl, 1 mM EDTA, pH = 8.0) to reach a buoyant density of 1.70 g mL^–1^. The mixed solution was transferred to 5 mL ultracentrifuge tubes, and subsequently centrifuged using a Beckman coulter Optima L-XP ultracentrifugation on a VTi 65.2 rotor (Beckman Coulter) at 179,000 *g* for 40 h at 20°C. Then, the gradients were fractionated into 14 fractions by displacement with distilled water using a syringe pump (NE-1000) at a flow rate of 780 μL min^–1^. The buoyant density of each fraction was measured with a digital refractometer (AR200 Digital Handheld Refractometer, United States). DNA in each fraction was precipitated with 100% ice ethanol and sodium acetate, washed with 70% ethanol, and dissolved in autoclaved Milli-Q water for further analysis.

### Separation of ^13^C- and ^12^C-DNA With Gradient Fractionation

The prokaryotic amplifier 515F (5′-GTGCCAGGMGCCG GGGTAA-3′)/907R (5′-CCGTCAATTCCTTTRAGTTT-3′) was used in a PCR to check the success of gradient fractionation from 1 to 14 (Supplementary Figure S2). The PCR program started with the initial denaturation at 95°C for 3 min, followed by 28 cycles of denaturation at 95°C for 30 s, annealing at 55°C for 30 s, and extension at 72°C for 45 s, with a final extension at 72°C for 10 min (Han et al., 2014). The DNA concentrations in Fractions 6, 7, 11, and 12 for all samples were indicated in Supplementary Table S1. Fractions 6 and 7 corresponded to heavy ^13^C-DNA fractions, and Fractions 8–14 as light ^12^C-DNA fractions. For the purpose of sequencing the substrate-metabolizing community, only Fractions 6 and 7 were analyzed.

### Illumina MiSeq Sequencing

The DNA (DNA without SIP) extracted from the soils sampled at Days 7, 14, and 28 (the entire community), and the ^13^C-DNA fractions at Days 7 and 28 (the community metabolizing residue) were subjected to Illumina Miseq sequencing. The V_4_-V_5_ regions of the 16S rRNA genes were amplified using the primer sets 515F and 907R. The amplification of each sample was performed twice, and the two PCR products were combined. An AxyPrepDNA purification kit (AXYGEN, Inc.) was used to purify PCR products. The purified amplicons (around 400 bp length fragments) were sent to Biozeron (Shanghai, China) for paired-end sequencing on an Illumina MiSeq platform (Caporaso et al., 2011, 2012).

### Analysis of Illumina Miseq Sequencing Data

Illumina MiSeq sequencing raw data were processed by the QIIME software package (Version 1.8.0) (Caporaso et al., 2010). For quality control, the reads were trimmed by discarding sequences of length shorter than 380 bp and quality scores lower than 20. In total, 667,192–1,338,771 high quality and chimera-free reads with an average length of 397 bp in each read were obtained throughout soil DNA samples and the ^13^C-DNA fractions, respectively. The random selection on the minimum reads was performed across all the samples. The operational taxonomic units (OTUs) were defined at 97% similarity level on sequences using Usearch (version 7.1^2^). The taxonomic identity of the phylotypes was assessed by the Ribosomal Database Project (RDP) Classifier (Version 2.2^3^) at a confidence threshold of 70%. All the sequences were uploaded onto the GenBank Sequence Read Archive (SRP141616 for DNA sequencing without SIP, SRP142322 for DNA sequencing of ^13^C-DNA fractions).

### Statistical Analysis

The statistical comparisons between the aCO_2_- and eCO_2_-derived residues were performed for C and N concentrations, C:N ratio and ^13^C abundances using Paired Student’s *t*-test at the 0.05 significance level (SPSS 20.0 for Windows).

The significance in α diversity of prokaryotic community and relative abundance at the genus level over time between the residue treatments was tested using the two-way ANOVA (GenStat, version 13.0, VSN International, Hemel Hemspstead, United Kingdom) and Duncan’s multiple range test at a 0.05 significance level (SPSS 20.0 for Windows). The data of the relative abundances of genera in ^13^C-DNA fractions were log-transformed to follow a normal distribution before variation analysis. Changes over time in prokaryotic community structure in response to residue amendments were analyzed using the principal co-ordinates analysis (PCoA) based on Bray-Curtis distance. The effect of residue amendment on prokaryotic community structure in ^13^C-DNA fractions over time were analyzed using PCoA based on Unweighted-Unifrac distance. The permutational multivariate ANOVA (PERMANOVA) was performed to assess the significance of difference in the microbial community structure between treatments. It was achieved by running the adonis function with the “vegan” package in the R version 3.3.1 for Windows (R Development Core Team, 2010; ADONIS, Oksanen et al., 2014).

## Results

### Chemical Properties of Residue

There was no significant difference in C or N concentration between the aCO_2_- and eCO_2_-derived residues (Table 1). The C/N ratio did not differ between the two treatments with an average of 12.7. Similarly, eCO_2_ did not significantly affect the concentration of cellulose in shoots. However, eCO_2_ decreased the concentration of lignin with a marginal significance. The ^13^C enrichment in the eCO_2_-derived residue was significantly higher than that in the aCO_2_-derived residue.

**TABLE 1:** Chemical properties of residues of soybean that were labeled with ^13^CO_2_ and exposed to aCO_2_ (390 ppm) or eCO_2_ (550 ppm) for 54 days.

**Treatments**	**C (g kg^–1^)**	**N (g kg^–1^)**	**C/N ratio**	**Cellulose (g kg^–1^)**	**Lignin (g kg^–1^)**	**δ^13^C abundance (‰)**	**Atom ^13^C%**
aCO_2_-derived residue	425 ± 2	35 ± 2	12.3 ± 0.6	299 ± 3	168 ± 12	24935 ± 464	22.5 ± 0.3
eCO_2_-derived residue	427 ± 1	33 ± 2	13.0 ± 1.0	292 ± 3	133 ± 3	40638 ± 1137	31.7 ± 0.6
Significance level (*p*)	0.843	0.794	0.275	0.300	0.053	<0.001	<0.001

**Data are mean ± standard error of three replicates.**

### α Diversity of Soil Prokaryotic Community in Response to Residue Amendments

The estimated diversity (as indicated by Shannon index) and richness (as indicated by Ace and Chao1 indices) of the entire prokaryotic community were 22 and 21% higher in the soil amended with eCO_2_-derived residue, compared to that with the aCO_2_-derived residue at Day 14, respectively (Table 2). There was no significant difference in the diversity or richness of the entire prokaryotic community between the two residue treatments at Days 7 and 28.

**TABLE 2:** Effects of residue type (aCO_2_- or eCO_2_-derived residues) and incubation time (Days) on α diversity indices (at 97% sequence similarity) of the whole soil prokaryotic community.

**Days**	**Residue**	**Shannon**	**Simpson**	**Ace**	**Chao1**	**Coverage**
7	aCO_2_-derived	1.91 ± 0.05 d	0.40 ± 0.01 a	190 ± 6 bc	191 ± 3 bc	0.997
	eCO_2_-derived	1.95 ± 0.27 cd	0.42 ± 0.07 a	202 ± 11 abc	203 ± 14 abc	0.997
14	aCO_2_-derived	2.21 ± 0.04 bcd	0.20 ± 0.01 b	185 ± 11 c	186 ± 10 c	0.997
	eCO_2_-derived	2.70 ± 0.08 a	0.14 ± 0.01 b	226 ± 4 a	225 ± 4 a	0.997
28	aCO_2_-derived	2.36 ± 0.04 abc	0.19 ± 0 b	211 ± 4 ab	214 ± 10 abc	0.997
	eCO_2_-derived	2.45 ± 0.11 ab	0.19 ± 0.01 b	217 ± 7 a	218 ± 3 ab	0.997
**Significance level (*p*)**						
Time		0.003	<0.001	0.120	0.129	–
Residue		0.074	0.640	0.010	0.019	–
Time × Residue		0.202	0.454	0.100	0.142	–

**The different letters within a column represent significant differences among the treatments (p < 0.05). Data were means ± standard error of three replicates.**

### Prokaryotic Community Structure in Response to Residue Amendments

**FIGURE 1:**
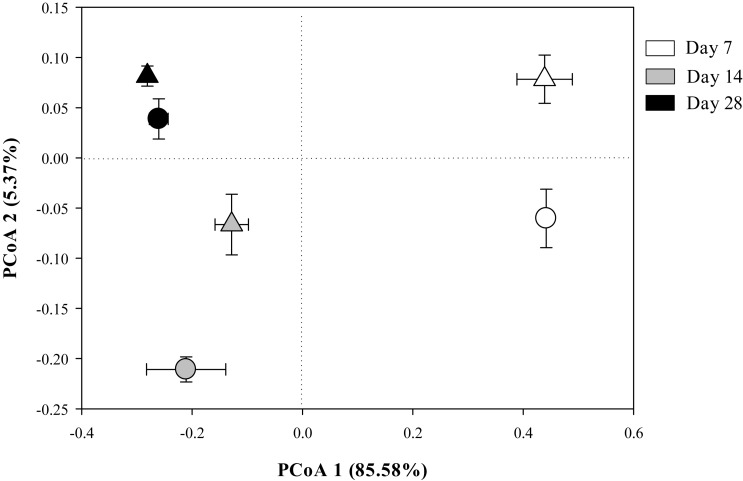
The principal coordinates analysis (PCoA) plot of the Bray-Curtis distances showing the whole prokaryotic community structure in Mollisols amended with aCO_2_- (circles) or eCO_2_-derived (triangles) soybean residues after 7, 14, and 28 days of incubation. Data were means ± standard error of three replicates.

The Bray-Curtis PCoA indicate that the entire prokaryotic community structure greatly changed over the incubation time (*p* < 0.05) (Table 3), but there was no significant difference in prokaryotic community structure at each sampling time point between the two residue treatments (Figure 1 and Table 3).

**TABLE 3:** Statistical summary for effects of residue type (aCO_2_- and eCO_2_-derived residues) and incubation time on the whole prokaryotic community structure tested by permutational multivariate analysis of variance (PERMANOVA) based on the Bray-Curtis distance metrics.

**Factors**	**SS**	**MS**	***F*-value**	**Significance level (*p*)**
Over time for aCO_2_-derived residue	0.951	0.475	57.568	0.004
Over time for eCO_2_-derived residue	1.000	0.500	37.646	0.011
Residue effect at Day 7	0.016	0.016	1.225	0.400
Residue effect at Day 14	0.042	0.042	3.692	0.100
Residue effect at Day 28	0.009	0.009	1.244	0.400

**The significance was assessed by F-tests from 999 permutations of the OTU data. Day 7, Days 14 and 28 represent 7, 14, and 28 days of incubation. SS and MS represent mean sum of squares and mean squares, respectively.**

**FIGURE 2:**
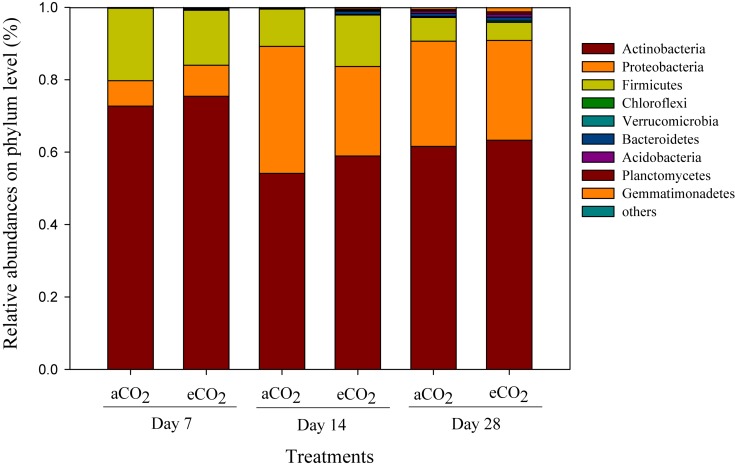
Taxonomic compositions of prokaryotic communities at the phylum level in the soils amended with soybean residues produced under aCO_2_ (390 ppm) or eCO_2_ (550 ppm) at Days 7, 14, and 28 after incubation. The aCO_2_ and eCO_2_ represent aCO_2_- and eCO_2_-derived residue amendments, respectively. Data were means ± standard error of three replicates.

Phyla including Acidobacteria (54.1–75.4%), Proteobacteria (7.0–35.1%), and Firmicutes (5.0–20.0%) were the dominant compositions of the soil prokaryotic community after amendment of soybean residues (Figure 2). The relative abundances of bacterial phyla, such as Actinobacteria, Proteobacteria, Firmicutes, and Verrucomicrobia significantly changed over the incubation period, but did not differ between the residue treatments (Figure 2).

For the genus level classification, we found that relative abundances of genera *Streptomyces*, *Bacillus*, and *Paenibacillus* decreased over time (*p* < 0.01), while that of *Xanthomonadaceae_unclassified* increased from Days 7 to 14 (*p* < 0.01) (Supplementary Table S2). The relative abundances of *Bryobacter*, *Microbispora*, *Steroidobacter*, *Bradyrhizobium*, and *Acetobacteraceae_unclassified* were higher, and that of *Rhizobiales_unclassified* was lower (*p* < 0.05) in the treatment of the eCO_2_-derived residue, compared with the aCO_2_-derived residue. However, their overall relative abundances were less than 0.5% (Supplementary Table S2).

### Prokaryotic Community Metabolizing Residue-C

**FIGURE 3:**
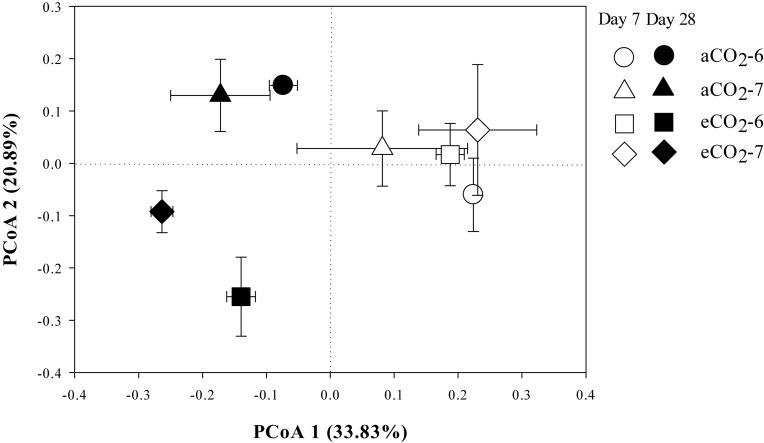
The principal coordinates analysis (PCoA) plot of the Unweighted-Unifrac distance showing the residue-metabolizing community structure in Mollisols amended with aCO_2_- or eCO_2_-derived soybean residues after 7 and 28 days of incubation. The aCO_2_ and eCO_2_ represent aCO_2_- and eCO_2_-derived residue amendments, and number 6 and 7 represent the 6th and 7th ^13^C-DNA fractions, respectively. Data were means ± standard error of three replicates.

The residue-metabolizing community structure was significantly (*p* = 0.001) different from the whole community (Supplementary Figure S2). The community structure of bacteria that metabolized residue greatly differed between the residue treatments 28 days after amendment (Figure 3 and Table 4). Genera *Streptomyces* belonging to phylum Actinobacteria, and *Bacillus* and *Terribacillus* belonging to phylum Firmicutes predominantly incorporated both residues (Table 5). The amendment of eCO_2_-derived residue led to lower relative abundances of *Actinomadura*, *Nocardia*, and *Shimazuella*, compared with the amendment of aCO_2_-derived residue. In contrast, the relative abundance of *Non-omuraea* was 87–90% higher in the treatment of eCO_2_-derived residue (Table 5).

**TABLE 4:** Statistical summary for the effect of residue type (aCO_2_- and eCO_2_-derived residues) on the community structure of residue-metabolizing bacteria at 7 and 28 days of incubation.

**Factors**	**SS**	**MS**	***F-*value**	**Significance level (*p*)**
Residue effect at Day 7	0.021	0.021	0.421	0.565
Residue effect at Day 28	0.336	0.336	7.857	0.015

**The data were tested by permutational multivariate analysis of variance (PERMANOVA) based on the Unweighted-Unifrac distance metrics. The significance was assessed by F-tests from 999 permutations of the OTU data. Days 7 and 28 represent 7 and 28 days of incubation. SS and MS mean sum of squares and mean squares, respectively.**

**TABLE 5:** Relative abundances (≥0.3%) of the dominant prokaryotic genera in the 6th and 7th fractions in DNA-SIP in response to the amendment of aCO_2_- or eCO_2_-derived soybean residues after 7 and 28 days of inoculation.

**Phylum**	**Genus**	**Day 7**				**Day 28**				**Significance level (*p*)**		
		**aCO_2_-6**	**aCO_2_-7**	**eCO_2_-6**	**eCO_2_-7**	**aCO_2_-6**	**aCO_2_-7**	**eCO_2_-6**	**eCO_2_-7**	**CO_2_**	**Days**	**CO_2_ × days**
Actinobacteria	*Actinomadura*	0.6 ± 0.6c	0.7 ± 0.6c	0 ± 0c	0 ± 0c	22.2 ± 4.1a	23.5 ± 2.6a	8.8 ± 0.5b	10.5 ± 0.4b	**<0.001**	**<0.001**	**<0.001**
	*Non-omuraea*	0.8 ± 0.8d	1.1 ± 0.7d	0 ± 0d	0 ± 0d	9.2 ± 0.9c	10.5 ± 1.0c	17.5 ± 1.8b	19.6 ± 0.5a	**<0.001**	**<0.001**	**<0.001**
	*Nocardia*	0.7 ± 0.7c	0.7 ± 0.7c	0 ± 0d	0 ± 0d	3.2 ± 0.4a	2.9 ± 0.2a	1.5 ± 0.1b	1.7 ± 0.4b	**<0.001**	**<0.001**	0.165
	*Streptomyces*	35.2 ± 0b	45.3 ± 5.8a	33.6 ± 9.2b	38.5 ± 6.7ab	16.5 ± 3.2d	19.1 ± 2.1cd	24.8 ± 2.4c	28.1 ± 1.9*b*c	0.446	**<0.001**	**0.049**
Firmicutes	*Bacillus*	45.4 ± 4.0a	44.5 ± 3.5a	43.2 ± 11.3ab	42.1 ± 6.8ab	43.9 ± 7.1ab	38.8 ± 1.8bc	40.5 ± 2.8b	35.9 ± 0.8c	0.438	0.314	0.967
	*Terribacillus*	8.2 ± 1.5^a^	7.8 ± 3.9a	10.3 ± 2.6a	9.1 ± 4.5a	3.3 ± 0.7b	3.9 ± 2.0b	1.0 ± 0.2b	0.9 ± 0.1b	0.728	**<0.001**	0.168
	*Shimazuella*	0.4 ± 0.4ab	0.5 ± 0.3a	0.2 ± 0b	0 ± 0b	0.6 ± 0.2a	0.5 ± 0.1a	0.2 ± 0.1b	0.1 ± 0b	**0.006**	0.529	0.682

**The different letters within a row represent significant differences among the treatments over time (p < 0.05). Data are mean ± standard error of three replicates. Number 6 and 7 behind aCO_2_ or eCO_2_ represent the 6th and 7th ^13^C-DNA fractions, respectively. Data were log(x + 100)-transformed before ANOVA. The p-values less than 0.05 were indicated in bold.**

There were no difference in the relative abundance of bacteria that incorporated residue-^13^C between the aCO_2_- and eCO_2_-derived residue amendments at Day 7 (Figure 3 and Table 4).

## Discussion

The entire structure of soil prokaryotic community as indicated by Bray-Curtis PCoA profile did not differ between the two residue treatments during 28 days incubation (Figure 1 and Table 3). This result was in accordance with our previous findings that compared with aCO_2_-derived residue, the amendment of eCO_2_-derived wheat residue did not significantly affect the entire prokaryotic community structure in the first 60 days of incubation (Wang et al., 2017).

However, the structure of bacteria community that utilized soybean residue was assumed to change over time (Supplementary Figure S3), indicating that residues specific select the soil community. In this study, two key time points, i.e., Days 7 and 28 were selected for SIP because the prokaryotic activity strongly respond to residue amendments during the early period of decomposition, especially the first 4 weeks (Bastian et al., 2009; Lian et al., 2017). Moreover, Bernard et al. (2007) indicated that using density gradient centrifugation, effective separation of labeled from non-labeled DNA could be achieved 7 days after amendment of a ^13^C-labeled wheat residue into a Calcarosol.

By Day 28, the composition of the bacteria assimilating the residues differed between the residues produced under different CO_2_ levels (Figure 2, Table 4, and Supplementary Table S2). Genera *Actinomadura*, *Non-omuraea*, *Nocardia*, and *Shimazuella* in the residue-metabolizing community were the key members that dominantly contributed to the difference (Table 5). Although genera *Nocardia* and *Shimazuella* were dominant residue-C consumers (Table 5), they were not detectable in the whole prokaryotic community (Supplementary Table S2). This observation implied that residues specifically selected residue-C consumers from the whole prokaryotic community reservoirs of the soil over time, and these consumers performed heir function on the residue-C turnover (Lian et al., 2017). This view was supported by significant difference between residue-metabolizing community and the whole community of soil (Supplementary Figure S2), and Blagodatskaya et al. (2010) showing that genus *Actinomadura* became a key member that was involved in the C turnover of *Brassica napus* residue, but this genus originally accounted for a very small proportion in the whole prokaryotic community in soil. Furthermore, the residue-C incorporating genus *Actinomadura* was almost twofold higher with the amendment of aCO_2_-derived residue than eCO_2_-derived residue (Table 5) while its relative abundance in the whole prokaryotic community had no difference between the two residue treatments (Supplementary Table S2). This suggests that residues derived from different CO_2_ environments distinctively shape the community of bacteria that metabolize residue-C much earlier than their effects on the entire community.

In particular, the lower relative abundances of *Actinomadura*, *Nocardia*, and *Shimazuella* in the eCO_2_-derived than the aCO_2_-derived residue treatment (Table 5) may be attributed to the residue chemistry. Although the residues derived from aCO_2_ and eCO_2_ conditions did not differ in most chemical properties, the eCO_2_-derived residue had marginally lower lignin level (*p* = 0.053) (Table 1). Therefore, it is plausible that the compositional changes in the prokaryotic community was attributed to the difference in lignin concentration and other unmeasured chemical properties. While *Nocardia* was able to utilize complex organic compounds (such as cellulose and trehalose) (Fang et al., 2017). The higher relative abundance of genus *Nocardia* in the aCO_2_-derived residue treatment soils suggested that this type of Actinobacteria might facilitate the decomposition of the complex compounds from the residual source. Meanwhile, genus *Shimazuella* can produce some metabolites such as hydroxyl and carbonyl (Boonsnongcheep et al., 2016), and some species affiliated to this genus are capable of degrading casein and starch (Park et al., 2007). Thus, the greater relative abundance of *Shimazuella* may favor starch degradation in the aCO_2_-derived residue, as the starch concentration in soybean shoot is usually higher under aCO_2_ compared to eCO_2_ (Bertrand et al., 2011).

In contrast, the relative abundance of the residue-metabolizing *Non-omuraea* was much higher in the eCO_2_-derived than the aCO_2_-derived residue. Genus *Non-omuraea* is classified as a slow-growing actinomycete taxon (Wang et al., 2011; Li et al., 2012), which differed from other members belong to phylum Actinobacteria. It is possible that *Non-omuraea* did not use the residue-C directly (by using the necromass from bacteria that assimilated plant residues) or it slowly degraded the residue-C for its own assimilation. The literature showed that *Non-omuraea* may degrade starch and guanine, and utilize sugars and amino acids as nitrogen source (Jose and Jebakumar, 2014). Moreover, this genus can produce antibiotics, which may kill other bacteria and produce secondary sources for its use (Sungthong and Nakaew, 2015). The various functions of *Non-omuraea* may be associated with residue properties that primarily affect its decomposition rate. However, it is unknown what sort of decomposable components in the eCO_2_-derived residue can be assessed by *Non-omuraea*.

Genera *Streptomyces* and *Bacillus* in the residue-metabolizing community were most abundant at the initial stage of incubation, indicating that these genera were potentially primary C sequesters consuming easily-decomposable C components. This is consistent with the findings of Semenov et al. (2012) and Eichorst et al. (2011) that *Streptomyces* and *Bacillus* were widely distributed in the ^13^C-DNA fractions of DNA in soils amended with ^13^C-labeled residues of soybean, maize and tomato. However, unlike *Actinomadura*, *Nocardia*, and *Non-omuraea*, genera *Streptomyces* and *Bacillus* showed similar responses to the amendment of the residues derived from aCO_2_ and eCO_2_ (Table 5). The species of *Streptomyces* were able to utilize various organic compounds, such as cellulose and lignin (Ulrich et al., 2008; Xu and Yang, 2010; España et al., 2011). Genus *Bacillus* is known as a proteolytic bacterium and is likely to be an important protease producer in soils (Watanabe and Hayano, 1994; Sakurai et al., 2007). The consistent enrichment of these genera over time may be associated with (i) the limited chemical variation between the residues derived from aCO_2_ and eCO_2_ treatments; (ii) the rapid turnover of their biomass, including the biosynthesis in microbes, microbial residue formation, and their reutilization. Indeed, the microbial products typically comprised exopolysaccharides, lipids, glycoproteins and peptidoglycan murein (Kögel-Knabner, 2017). The relative importance of these products in regulation of the residue-assimilating prokaryotic community needs to be explored by analyzing ^13^C-labeled microbial products and their correlations with the potential consumers.

## Conclusion

The amendments of eCO_2_-derived soybean residue triggered the different response in prokaryotic community structure compared with aCO_2_, though major chemical characteristics of residues were not statistically different between the two CO_2_ treatments. Genera in phyla Actinobacteria and Firmicutes were the major members that assimilate soybean residue. Residue-assimilating genus *Non-omuraea* was enriched in the amendment of eCO_2_-derived residue, while genera *Actinomadura*, *Nocardia*, and *Shimazuella* showed the opposite after 28 days of incubation. The residue source did not affect the whole community structure of bacteria during the period of incubation. The community of C-metabolizing bacteria responded earlier to residue amendments than the whole soil prokaryotic community. It is worthy to further identify original plant residue-C compounds and their biosynthesized products in microbes linking with microbial community composition in the future study.

## Data Availability Statement

Publicly available datasets were analyzed in this study. This data can be found here: https://www.ncbi.nlm.nih.gov/sra/?term=SRP141616 for DNA sequencing without SIP, and https://www.ncbi.nlm.nih.gov/sra/?term=SRP142322 for DNA sequencing of 13C-DNA fractions.

## Author Contributions

YW, ZY, and YL performed the experiment. ZY, JL, and ZX analyzed the data. GW, XL, and JJ designed the experiment. CT and JJ discussed the results and wrote up the manuscript.

## Conflict of Interest

The authors declare that the research was conducted in the absence of any commercial or financial relationships that could be construed as a potential conflict of interest.

## Footnotes

1 https://climate.nasa.gov

2 http://drive5.com/uparse/

3 http://sourceforge.net/projects/rdp-classifier/

## References

[B1] Ainsworth E. A. Leakey A. D. B. Ort D. R. Long S. P. (2008). FACE-ing the facts: inconsistencies and interdependence among field, chamber and modeling studies of elevated [CO_2_] impacts on crop yield and food supply. *New Phytol.* 179 5–9. 10.1111/j.1469-8137.2008.02500.x 18482226

[B2] Ainsworth E. A. Long S. P. (2005). What have we learned from 15 years of free-air CO_2_ enrichment (FACE)? a meta-analytic review of the responses of photosynthesis, canopy properties and plant production to rising CO_2_. *New Phytol.* 165 351–372. 10.1111/j.1469-8137.2004.01224.x 15720649

[B3] Bastian F. Bouziri L. Nicolardot B. Ranjard L. (2009). Impact of wheat straw decomposition on successional patterns of soil microbial community structure. *Soil Biol. Biochem.* 41 262–275. 10.1016/j.soilbio.2008.10.024

[B4] Baumann K. Marschner P. Smernik R. J. Baldock J. A. (2009). Residue chemistry and microbial community structure during decomposition of eucalypt, wheat and vetch residues. *Soil Biol. Biochem.* 41 1966–1975. 10.1016/j.soilbio.2009.06.022

[B5] Bernard L. Mougel C. Maron P. A. Nowak V. Lévêque J. Henault C. (2007). Dynamics and identification of soil microbial populations actively assimilating carbon from ^13^C-labelled wheat residue as estimated by DNA- and RNA-SIP techniques. *Environ. Microb.* 9 752–764. 10.1111/j.1462-2920.2006.01197.x 17298374

[B6] Bertrand A. Prévost D. Juge C. Chalifour F. P. (2011). Impact of elevated CO_2_ on carbohydrate and ureide concentrations in soybean inoculated with different strains of *Bradyrhizobium japonicum*. *Botany* 89 481–490. 10.1139/b11-034

[B7] Blagodatskaya E. Blagodatsky S. Dorodnikov M. Kuzyakov Y. (2010). Elevated atmospheric CO_2_ increases microbial growth rates in soil: results of three CO_2_ enrichment experiments. *Glob. Change Biol.* 16 836–848. 10.1111/j.1365-2486.2009.02006.x

[B8] Boonsnongcheep P. Nakashima T. Takahashi Y. Prathanturarug S. (2016). Diversity of endophytic actinomycetes isolated from roots and root nodules of *Pueraria candollei* grah. ex benth. and the analyses of their secondary metabolites. *Chiang Mai J. Sci.* 44 1–14.

[B9] Butterly C. R. Phillips L. A. Wiltshire J. L. Franks A. E. Armstrong R. D. Chen D. (2016). Long-term effects of elevated CO_2_ on carbon and nitrogen functional capacity of microbial communities in three contrasting soils. *Soil Biol. Biochem.* 97 157–167. 10.1016/j.soilbio.2016.03.010

[B10] Caporaso J. G. Bittinger K. Bushman F. D. Desantis T. Z. Andersen G. L. Knight R. (2010). PyNAST?: a flexible tool for aligning sequences to a template alignment. *Bioinformatics* 26 266–267. 10.1093/bioinformatics/btp636 19914921 PMC2804299

[B11] Caporaso J. G. Lauber C. L. Walters W. A. Berg-Lyons D. Huntley J. Fierer N. (2012). Ultra-high-throughput microbial community analysis on the illumina hiseq and MiSeq platforms. *ISME J.* 6 1621–1624. 10.1038/ismej.2012.8 22402401 PMC3400413

[B12] Caporaso J. G. Lauber C. L. Walters W. A. Berg-Lyons D. Lozupone C. A. Turnbaugh P. J. (2011). Global patterns of 16S rRNA diversity at a depth of millions of sequences per sample. *Proc. Natl. Acad. Sci. U.S.A.* 108(Suppl. l), 4516–4522. 10.1073/pnas.1000080107 20534432 PMC3063599

[B13] Cotrufo M. F. Ineson P. (2000). Does elevated atmospheric CO_2_ concentrations affect wood decomposition? *Plant Soil* 224 51–57. 10.1023/a:1004771426605

[B14] de Graaff M. A. van Groenigen K. J. Six J. Hungate B. van Kessel C. (2006). Interactions between plant growth and soil nutrient cycling under elevated CO_2_: a meta-analysis. *Glob. Change Biol.* 12 2077–2091. 10.1111/j.1365-2486.2006.01240.x

[B15] Drenovsky R. E. Vo D. Graham K. J. Scow K. M. (2004). Soil water content and organic carbon availability are major determinants of soil microbial community composition. *Microb. Ecol.* 48 424–430. 10.1007/s00248-003-1063-2 15692862

[B16] Eichorst S. A. Kuske C. R. Schmidt T. M. (2011). Influence of plant polymers on the distribution and cultivation of bacteria in the phylum acidobacteria. *Appl. Environ. Microb.* 77 586–596. 10.1128/AEM.01080-10 21097594 PMC3020536

[B17] España M. Rasche F. Kandeler E. Brune T. Rodriguez B. Bending G. D. (2011). Identification of active bacteria involved in decomposition of complex maize and soybean residues in a tropical Vertisol using ^15^N-DNA stable isotope probing. *Pedobiologia* 54 187–193. 10.1016/j.pedobi.2011.03.001

[B18] Fan F. Yin C. Tang Y. Li Z. Song A. Wakelin S. A. (2014). Probing potential microbial coupling of carbon and nitrogen cycling during decomposition of maize residue by 13C-DNA-SIP. *Soil Biol. Biochem.* 70 12–21. 10.1016/j.soilbio.2013.12.002

[B19] Fang B. Z. Salam N. Han M. X. Jiao J. Y. Cheng J. Wei D. Q. (2017). Insights on the effects of heat pretreatment, ph, and calcium salts on isolation of rare *Actinobacteria* from karstic caves. *Front. Microbiol.* 8:1535. 10.3389/fmicb.2017.01535 28848538 PMC5550672

[B20] FAO-UNESCO (1974). *Soil Map of the World 1:5000000.* Paris: UNESCO.

[B21] Gifford R. M. Barrett D. J. Lutze J. L. (2000). The effects of elevated [CO_2_] on the C:N and C:P mass ratios of plant tissues. *Plant Soil* 224 1–14. 10.1023/A:1004790612630

[B22] Goering H. K. van Soest P. J. (1970). *Forage Fibre Analyses (Apparatus, Reagent, Procedure and Some Applications).* Washington, DC: Agricultural Handbook.

[B23] Han S. Chen Y. Hu J. Ji Z. (2014). Tongue images and tongue coating microbiome in patients with colorectal cancer. *Microb. Pathog.* 77 1–6. 10.1016/j.micpath.2014.10.003 25281933

[B24] Jose A. P. Jebakumar S. R. D. (2014). Successive nonstatistical and statistical approaches for the improved antibiotic activity of rare actinomycete *Nonomuraea* sp. JAJ18. *Biomed. Res. Int.* 240:906097. 10.1155/2014/906097 25276828 PMC4168032

[B25] Kögel-Knabner I. (2017). The macromolecular organic composition of plant and microbial residues as inputs to soil organic matter: fourteen years on. *Soil Biolog. Biochem.* 105 A3–A8. 10.1016/j.soilbio.2016.08.011

[B26] Lee C. G. Watanabe T. Murase J. Asakawa S. Kimura M. (2012). Growth of methanogens in an oxic soil microcosm: elucidation by a DNA-SIP experiment using ^13^C-labeled dried rice callus. *Appl. Soil Ecol.* 58 37–44. 10.1016/j.apsoil.2012.03.002

[B27] Li X. Zhang L. Ding Y. Gao Y. Ruan J. Huang Y. (2012). *Nonomuraea jiangxiensis* sp. nov., isolated from acidic soil. *Int. J. Syst. Evol. Micr.* 62 1409–1413. 10.1099/ijs.0.034520-0 21828009

[B28] Li Y. Yu Z. Liu X. Mathesius U. Wang G. Tang C. (2017). Elevated CO_2_ increases nitrogen fixation at the reproductive phase contributing to various yield responses of soybean cultivars. *Front. Plant Sci.* 8:1546. 10.3389/fpls.2017.01546 28959266 PMC5603704

[B29] Lian T. Jin J. Wang G. Tang C. Yu Z. Li Y. (2017). The fate of soybean residue-carbon links to changes of bacterial community composition in Mollisols differing in soil organic carbon. *Soil Biol. Biochem.* 109 50–58. 10.1016/j.soilbio.2017.01.026

[B30] Ma H. L. Zhu J. G. Xie Z. B. Liu G. Zeng Q. (2009). Effects of increased residue biomass under elevated CO_2_ on carbon and nitrogen in soil aggregate size classes (rice-wheat rotation system, China). *Can. J. Soil Sci.* 89 567–577. 10.4141/CJSS08049

[B31] Nguyen T. T. Cavagnaro T. R. Ngo H. T. T. Marschner P. (2016). Soil respiration, microbial biomass and nutrient availability in soil amended with high and low C/N residue-Influence of interval between residue additions. *Soil Biol. Biochem.* 95 189–197. 10.1016/j.soilbio.2015.12.020

[B32] Norby R. J. Cortufo M. F. Ineson P. O’Neill E. G. Canadell J. G. (2001). Elevated CO_2_, litter chemistry, and decomposition: a synthesis. *Oecologia* 127 153–165. 10.1007/s004420000615 24577644

[B33] Nowak R. S. Ellsworth D. S. Smith S. D. (2004). Functional responses of plants to elevated atmospheric CO_2_- do photosynthetic and productivity data from FACE experiments support early prediction? *New Phytol.* 162 253–280. 10.1111/j.1469-8137.2004.01033.x

[B34] Oksanen J. Blanchet F. G. Kindt R. Legendre P. Minchin P. R. O’Hara R. B. (2014). *Vegan**: Community Ecology Package.* Available at: http://CRAN.R-project.org/package=vegan (accessed March 19, 2018).

[B35] Park D. J. Dastager S. G. Lee J. C. Yeo S. H. Yoon J. H. (2007). *Shimazuella kribbensis* gen. nov., sp nov., a mesophilic representative of the family Thermoactinomycetaceae. *Int. J. Syst. Evol. Micr.* 57:2660. 10.1099/ijs.0.65194-0 17978236

[B36] Pascault N. Ranjard L. Kaisermann A. Bachar D. Christen R. Terrat S. (2013). Stimulation of different functional groups of bacteria by various plant residues as a driver of soil priming effect. *Ecosystems* 16 810–822. 10.1007/s10021-013-9650-7

[B37] R Development Core Team (2010). *R: A Language and Environment for Statistical Computing.* Vienna: R Foundation for Statistical Computing.

[B38] Radajewski S. Ineson P. Parekh N. R. Murrell J. C. (2000). Stable-isotope probing as a tool in microbial ecology. *Nature* 403 646–649. 10.1038/35001054 10688198

[B39] Rui J. P. Peng J. J. Lu Y. H. (2009). Succession of bacterial populations during plant residue decomposition in rice field soil? *Appl. Environ. Microb.* 75 4879–4886. 10.1128/AEM.00702-09 19465536 PMC2708425

[B40] Sakurai M. Suzuki K. Onodera M. Shinano T. Osaki M. (2007). Analysis of bacterial communities in soil by PCR-DGGE targeting protease genes. *Soil Biol. Biochem.* 39 2777–2784. 10.1016/j.soilbio.2007.05.026

[B41] Sayer E. J. Heard M. S. Grant H. K. Marthews T. R. Tanner E. V. J. (2011). Soil carbon release enhanced by increased tropical forest litterfall. *Nat. Clim. Chang.* 1 304–307. 10.1038/nclimate1190

[B42] Semenov A. V. e Silva M. C. P. Szturc-Koestsier A. E. Schmitt H. Salles J. F. van Elsas J. D. (2012). Impact of incorporated fresh ^13^C potato tissues on the bacterial and fungal community composition of soil. *Soil Biol. Biochem.* 49 88–95. 10.1016/j.soilbio.2012.02.016

[B43] Sulman B. N. Phillips R. P. Oishi A. C. Shevliakova E. Pacala S. W. (2014). Microbe-driven turnover offsets mineral-mediated storage of soil carbon under elevated CO_2_. *Nat. Clim. Chang.* 4 1099–1102. 10.1038/nclimate2436

[B44] Sul W. J. Park J. Quensen J. F. Rodrigues J. L. Seliger L. Tsoi T. V. (2009). DNA-stable isotope probing integrated with metagenomics for retrieval of biphenyl dioxygenase genes from polychlorinated biphenyl-contaminated river sediment. *Appl. Environ. Microb.* 75 5501–5506. 10.1128/AEM.00121-09 19648381 PMC2737913

[B45] Sungthong R. Nakaew N. (2015). The genus *Nonomuraea*: a review of a rare actinomycete taxon for novel metabolites. *J. Basic Microb.* 55:554. 10.1002/jobm.201300691 24633812

[B46] Torbert H. A. Prior S. A. Rogers H. H. Wood C. W. (2000). Review of elevated atmospheric CO_2_ effects on agro-ecosystems: residue decomposition processes and soil C storage. *Plant Soil* 224 59–73. 10.1023/A:1004797123881

[B47] Ulrich A. Klimke G. Wirth S. (2008). Diversity and activity of cellulose-decomposing bacteria, isolated from sandy and loamy soil after long-term manure application. *Microb. Ecol.* 55 512–522. 10.1007/s00248-007-9296-0 17665240

[B48] van Groenigen K. J. Qi X. Osenberg C. W. Luo Y. Q. Hungate B. A. (2014). Faster decomposition under increased atmospheric CO_2_ limits soil carbon storage. *Science* 344 508–509. 10.1126/science.1249534 24762538

[B49] Viswanath T. Pal D. Purakayastha T. J. (2010). Elevated CO_2_ reduces rate of decomposition of rice and wheat residues in soil. *Agric. Ecosyst. Environ.* 139 557–564. 10.1016/j.agee.2010.09.016

[B50] Wang F. Xu X. X. Qu Z. Wang C. Lin H. P. Xie Q. Y. (2011). *Nonomuraea wenchangensis* sp. nov., isolated from mangrove rhizosphere soil. *Int. J. Syst. Evol. Micr.* 61 1304–1308. 10.1099/ijs.0.025742-0 20639224

[B51] Wang Y. H. Yu Z. H. Li Y. S. Wang G. H. Liu J. J. Liu J. D. (2017). Microbial association with the dynamics of particulate organic carbon in response to the amendment of elevated CO_2_-derived wheat residue into a Mollisol. *Sci. Total. Environ.* 60 972–981. 10.1016/j.scitotenv.2017.07.087 28724229

[B52] Watanabe K. Hayano K. (1994). Estimate of the source of soil protease in upland fields. *Biol. Fertil. Soils* 18 341–346. 10.1007/BF00570638

[B53] Williams M. A. Rice C. W. Owensby C. E. (2000). Carbon dynamics and microbial activity in tallgrass prairie exposed to elevated CO_2_ for 8 years. *Plant Soil* 227 127–137. 10.1023/A:1026590001307

[B54] Xu J. Yang Q. (2010). Isolation of rice straw degrading *Streptomyces griseorubens* C-5. *Biodegradation* 21 107–116. 10.1007/s10532-009-9285-8 19597946

[B55] Yu Z. Li Y. Jin J. Liu X. Wang G. (2017). Carbon flow in the plant-soil-microbe continuum at different growth stages of maize grown in a Mollisol. *Arch. Agron. Soil Sci.* 63 362–374. 10.1080/03650340.2016.1211788

