# Supplementary material for: 13C-DNA-SIP Distinguishes the Prokaryotic Community That Metabolizes Soybean Residues Produced Under Different CO2 Concentrations
Source: Front Microbiol. 2019 Sep 24;10:2184. doi: 10.3389/fmicb.2019.02184 (PMC6798031; doi:10.3389/fmicb.2019.02184)
Supplement: Supplementary file 1 [file Data_Sheet_1.pdf]

SUPPLEMENTARY MATERIAL

TABLE S1 Concentrations of DNA in various SIP fractions from aCO_2- and eCO_2-derived residues after 7 and 28 days of incubation.

Days	Residue	Replicates	Fractions (ng μL^{-1})			
			6	7	11	12
Day 7	aCO_2	I	0.38	6.10	5.11	8.09
		II	0.59	5.86	7.22	8.44
		III	1.11	5.90	7.19	6.45
	eCO_2	I	5.45	6.58	5.55	4.64
		II	6.15	6.56	5.56	7.06
		III	5.75	7.24	7.05	7.10
Day 28	aCO_2	I	7.97	6.07	4.67	3.64
		II	5.61	4.29	3.95	4.14
		III	3.81	6.17	3.57	4.12
	eCO_2	I	5.15	4.89	3.87	2.86
		II	6.15	4.74	3.55	2.91
		III	4.06	6.08	2.96	4.84

TABLE S2 Relative abundances (≥ 0.3%) of the dominant bacterial genera of the whole bacterial community in response to the amendment of aCO_2- or eCO_2-derived soybean residues after 7, 14 and 28 days of inoculation

Phylum	Genus	Day 7		Day 14		Day 28		LSD	Significance level (p)		
		aCO_2	eCO_2	aCO_2	eCO_2	aCO_2	eCO_2	(p=0.05)	CO_2	days	CO_2×days
Acidobacteria	*Bryobacter*	0 ± 0	0 ± 0	0.1 ± 0	0.2 ± 0	0.6 ± 0.1	0.7 ± 0.1	0.100	**0.042**	**<0.001**	0.375
Actinobacteria	*Nonomuraea*	1.2 ± 0.1	1.0 ± 0.3	30.5 ± 2.9	28.9 ± 1.0	38.1 ± 1.9	43.0 ± 2.0	3.023	0.507	**<0.001**	0.221
	Streptomyces	64.4 ± 0.2	67.1 ± 5.8	15.3 ± 2.0	19.2 ± 2.0	12.6 ± 1.0	11.2 ± 0.1	4.750	0.446	**<0.001**	0.599
	Actinomadura	1.1 ± 0.1	0.9 ± 0.1	6.0 ± 0.2	5.1 ± 0.5	7.6 ± 1.4	6.2 ± 0.3	1.127	0.117	**<0.001**	0.621
	Micromonospora	0.9 ± 0.1	1.0 ± 0.4	0.8 ± 0.2	1.7 ± 0.6	0.7 ± 0.1	1.1 ± 0.1	0.548	0.083	0.527	0.378
	Intrasporangiaceae_unclassified	1.8 ± 0.1	2.1 ± 0.7	0.3 ± 0.1	1.4 ± 0.6	0.7 ± 0.1	0.8 ± 0	0.650	0.111	**0.010**	0.395
	Pseudarthrobacter	1.9 ± 0	1.8 ± 0.5	0.1 ± 0.1	0.7 ± 0.2	0.3 ± 0	0.3 ± 0	0.367	0.329	**<0.001**	0.235
	Microbispora	0 ± 0	0 ± 0	0.1 ± 0	0.2 ± 0	0.3 ± 0	0.3 ± 0	0.031	**0.004**	**<0.001**	0.056
Bacteroidetes	*Chitinophaga*	0 ± 0	0 ± 0	0.1 ± 0.1	0.5 ± 0.2	0.6 ± 0.2	0.7 ± 0.1	0.247	0.150	**0.002**	0.385
Firmicutes	*Bacillus*	11.1 ± 1.4	5.3 ± 0.8	7.7 ± 1.7	11.5 ± 0.8	5.0 ± 0.1	3.9 ± 0.1	1.828	0.232	**<0.001**	**0.002**
	Paenibacillus	6.5 ± 1.4	7.5 ± 1.0	1.9 ± 0.9	2.1 ± 0.2	1.1 ± 0.4	0.9 ± 0.1	1.452	0.645	**<0.001**	0.720
	Cohnella	0 ± 0.2	0.1 ± 0.2	0.1 ± 0.4	0 ± 0	0 ± 0	0 ± 0	0.191	0.930	0.289	0.687
Gemmatimonadetes	*Gemmatimonadaceae_norank*	0 ± 0	0.1 ± 0	0 ± 0	0.1 ± 0	0.4 ± 0.1	0.8 ± 0.2	0.171	0.084	**<0.001**	0.196
Planctomycetes	*Singulisphaera*	0 ± 0	0 ± 0	0 ± 0	0.1 ± 0	0.2 ± 0.1	0.3 ± 0.1	0.092	0.244	**0.001**	0.684
Proteobacteria	*Xanthomonadaceae_unclassified*	3.6 ± 0.3	4.8 ± 3.8	30.4 ± 3.1	18.9 ± 3.3	24.6 ± 3.8	22.0 ± 1.4	5.230	0.101	**<0.001**	0.130
	Frateuria	0.1 ± 0	0.1 ± 0.1	0.4 ± 0.1	0.4 ± 0.1	0.6 ± 0.2	0.5 ± 0.1	0.187	0.982	**0.001**	0.786
	Rhizobiales_unclassified	0.8 ± 0.1	0.5 ± 0.1	0.5 ± 0	0.3 ± 0	0.5 ± 0.1	0.2 ± 0.1	0.142	**<0.001**	**0.007**	0.836
	Roseomonas	0± 0	0.1 ± 0	0.4 ± 0.2	0.3 ± 0	0.4 ± 0.1	0.4 ± 0.1	0.161	0.743	**0.008**	0.584
	Steroidobacter	0 ± 0	0 ± 0	0.2 ± 0.1	0.8 ± 0.1	0.3 ± 0.1	0.4 ± 0	0.136	**0.005**	**<0.001**	**0.008**
	Microvirga	1.1 ± 0.2	1.0 ± 0.4	0.8 ± 0.1	0.9 ± 0.1	0.3 ± 0.1	0.7 ± 0.1	0.335	0.424	**0.039**	0.505
	Bradyrhizobium	0.1 ± 0	0.2 ± 0.1	0.1 ± 0	0.4 ± 0	0.3 ± 0.1	0.4 ± 0.1	0.109	**0.013**	**0.034**	0.276
	Phenylobacterium	0.1 ± 0	0.2 ± 0.1	1.0 ± 0.1	0.4 ± 0	0.4 ± 0	0.4 ± 0.1	0.155	0.076	**<0.001**	**0.002**
	Acetobacteraceae_unclassified	0 ± 0	0.1 ± 0	0.2 ± 0.1	0.3 ± 0	0.2 ± 0.1	0.4 ± 0.1	0.107	**0.046**	**0.001**	0.542
	Sphingomonas	0.1 ± 0	0.1 ± 0.1	0.1 ± 0.1	0.2 ± 0	0.2 ± 0	0.2 ± 0	0.089	0.131	0.117	0.370
	Proteobacteria_unclassified	0 ± 0	0 ± 0	0.1 ± 0.1	0.3 ± 0	0.2 ± 0	0.2 ± 0.1	0.071	0.105	**<0.001**	0.075

Data are means ± standard error of three replicates. The p values less than 0.05 were highlighted in bold.

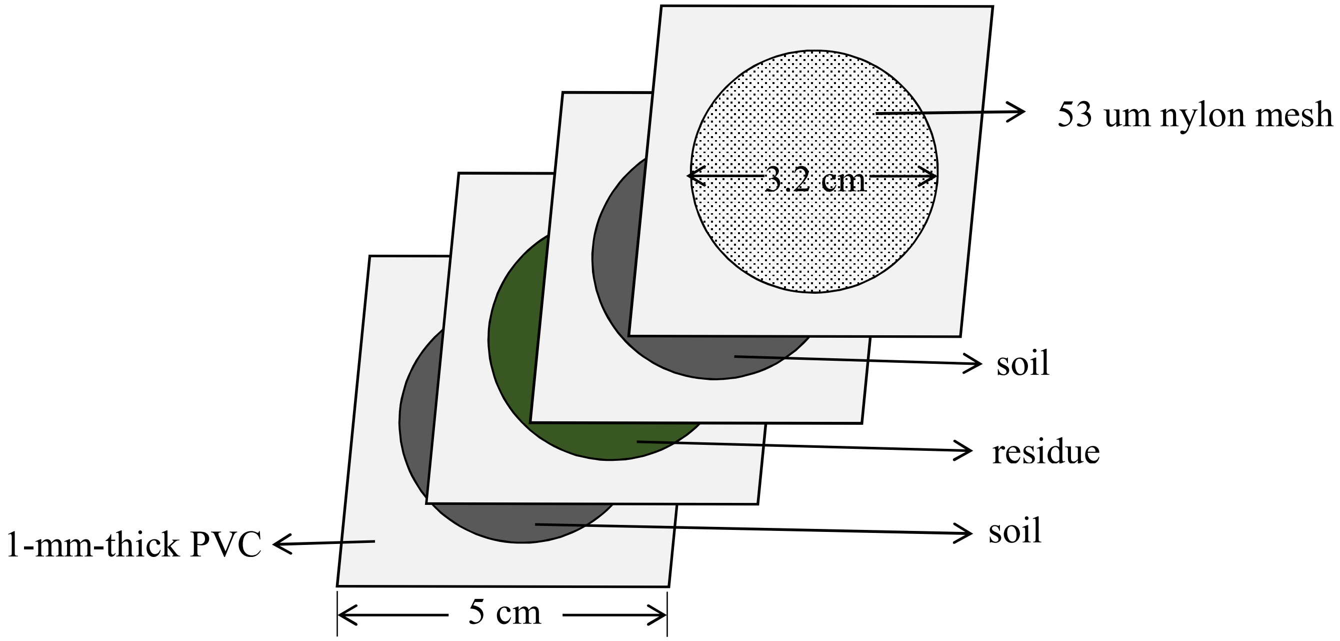

FIGURE S1 A schematic diagram of the three-compartment microcosm

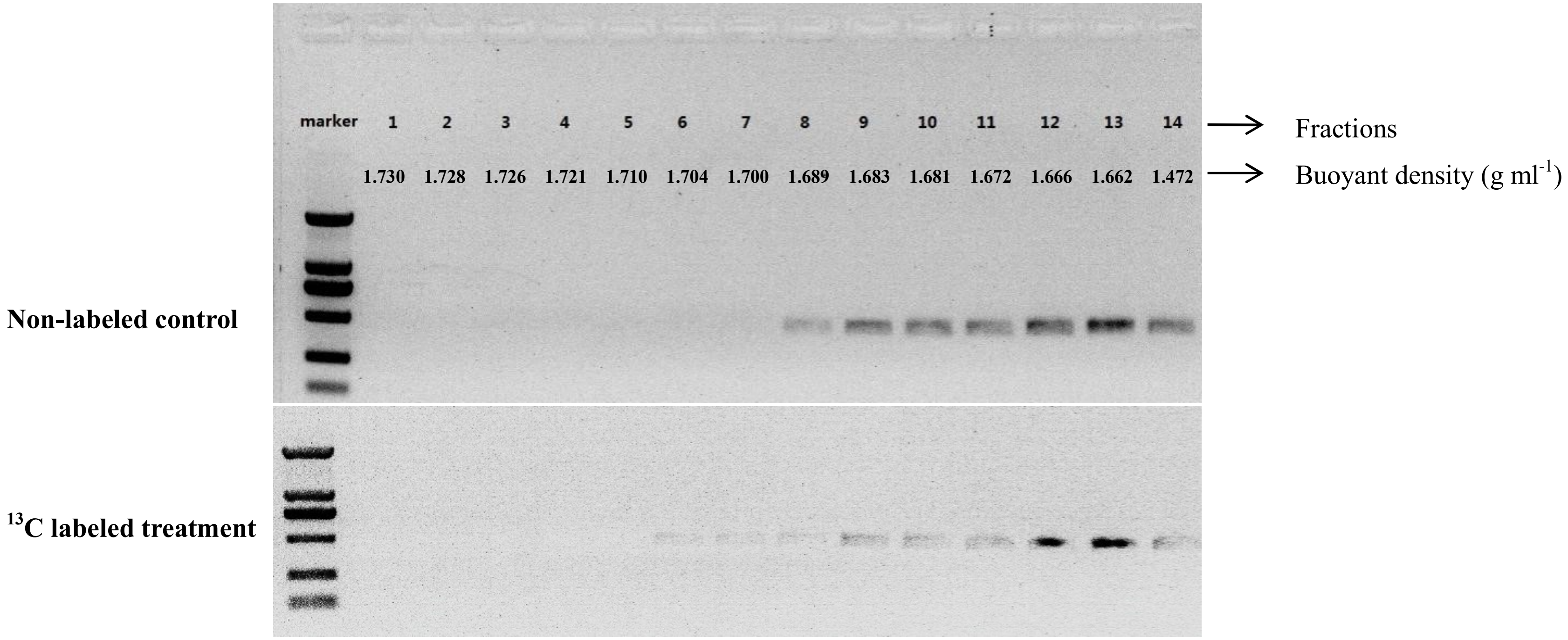

FIGURE S2 Aliquots of gradient fractions of DNA-SIP checked on 1% agarose gels and a 2000-bp ladder was included as a marker. Marker bands from the top to the bottom refer to 2000, 1000, 750, 500, 250 and 100 bp, respectively.

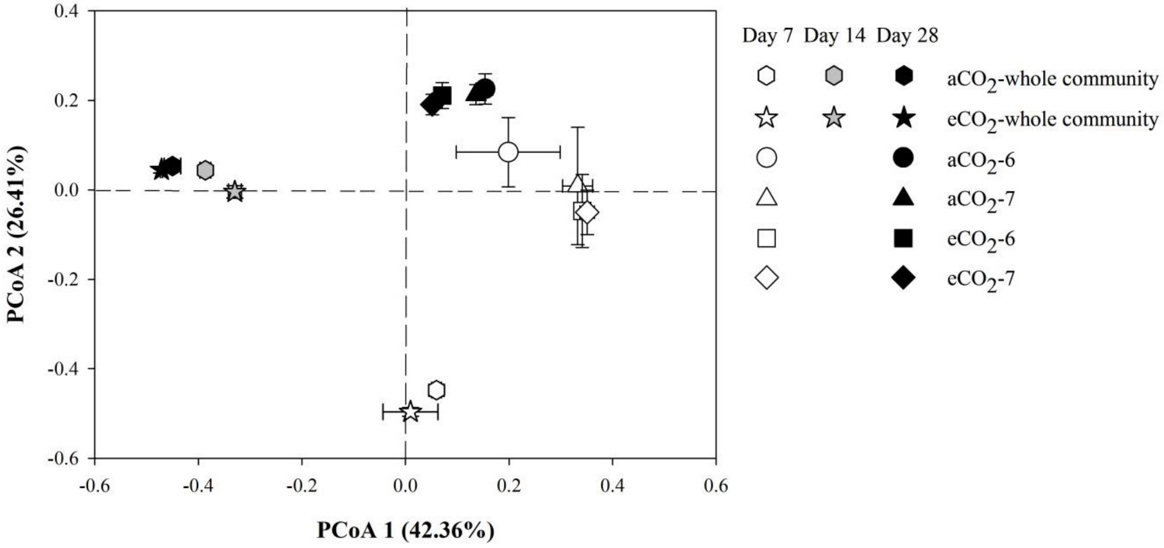

FIGURE S3. The principal coordinates analysis (PCoA) plot of the Bray-Curtis distances showing the dynamics of the whole and residue-metabolizing prokaryotic community structure in Mollisols amended with aCO_2- or eCO_2-derived soybean residues after 7, 14 and 28 days of incubation. The aCO_2 and eCO_2 represent aCO_2- and eCO_2-derived residue amendments, and number 6 and 7 represent the 6th and 7th ^{13}C-DNA fractions, respectively. Data were mean of three replicates ± standard error.
